# New-onset psoriasis with abrocitinib therapy for atopic dermatitis

**DOI:** 10.1016/j.jdcr.2025.04.002

**Published:** 2025-04-14

**Authors:** Sarah L. Becker, Adeeb Haroon, Eric Simpson

**Affiliations:** Department of Dermatology, Oregon Health and Science University, Portland, Oregon

**Keywords:** abrocitinib, atopic dermatitis, JAK inhibitor, psoriasiform eruption, psoriasis

## Introduction

Atopic dermatitis (AD) is one of the most common inflammatory skin conditions affecting up to 10% of adults and 25% of children worldwide.^1^^,^^2^ AD is a heterogenous disease with a wide spectrum of clinical phenotypes and variable responses to therapies. Janus kinase (JAK) inhibitors have been a promising development in the treatment of AD, particularly for patients who fail to respond to biologics. Abrocitinib is a JAK inhibitor which primarily inhibits JAK1 leading to suppression of inflammatory cytokines including interleukins (ILs) 4, 13, and 31 which have been implicated in the pathogenesis of AD.^3^ Abrocitinib is generally well tolerated, with the most common adverse events similar to those of other JAK inhibitors including nausea, upper respiratory tract infection, and headache. Targeted systemic treatments for chronic cutaneous immune-mediated diseases (IMDs), such as AD, may rarely cause the development of a second chronic IMD.^4^^,^^5^ While most cases of drug-related new onset cutaneous IMDs involve the targeting of 1 cytokine, there are recent reports of broader acting immune modulators, specifically the JAK inhibitor upadacitinib used for AD, leading to psoriasis.^5^ Herein, we report a case of a patient who developed psoriasis after initiating treatment with abrocitinib for AD.

## Case report

**Fig 1:**
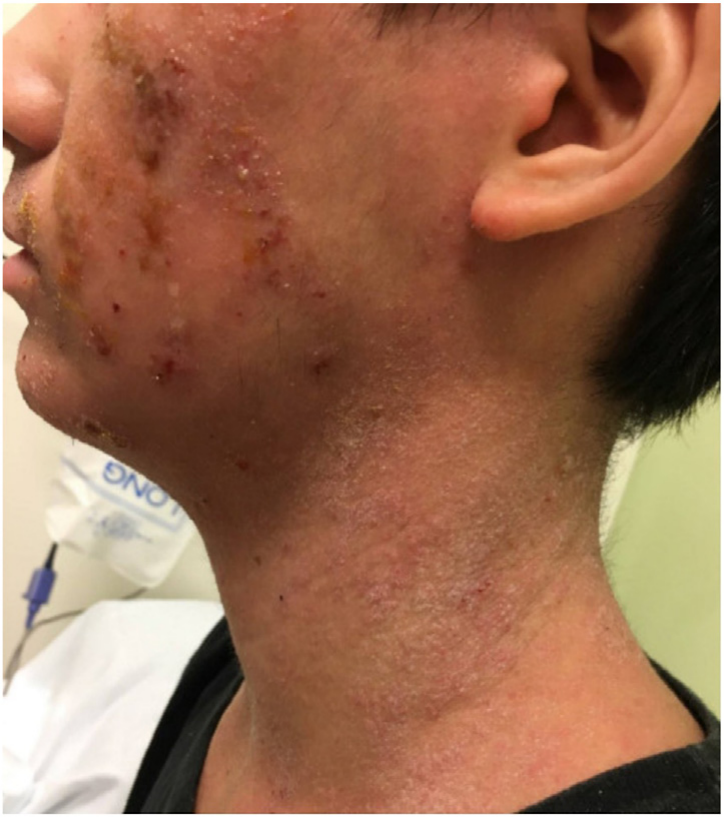
Eczematous dermatitis. The patient initially presented with a history of severe, recalcitrant atopic dermatitis with involvement most prominently of his head and neck with evidence of lichenified plaques consistent with chronic atopic dermatitis.

The patient is a 20-year-old male with a history of severe AD which initially involved the flexural surfaces in childhood but progressed to erythrodermic, generalized AD during adolescence (Fig 1). The patient has a personal history of asthma, seasonal and food allergies, and a family history of asthma and AD. Previous treatments for his AD included topical steroids, topical nonsteroids, prednisone, cyclosporine, methotrexate, and narrow-band ultraviolet light all with incomplete responses. A 6-month trial of dupilumab failed to show adequate clinical improvement and was discontinued due to development of acute pancreatitis. The patient was subsequently transitioned to abrocitinib.

**Fig 2:**
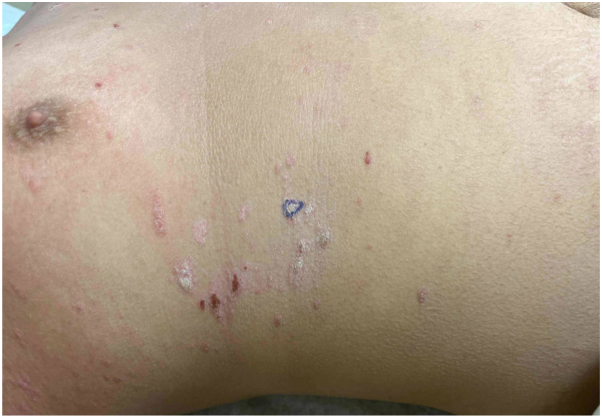
Psoriasiform dermatitis. While on abrocitinib, the patient developed an eruption of scaly, salmon-colored, well-demarcated papules and plaques affecting the trunk. Shown is the biopsy site which demonstrated transformation to psoriasis.

**Fig 3:**
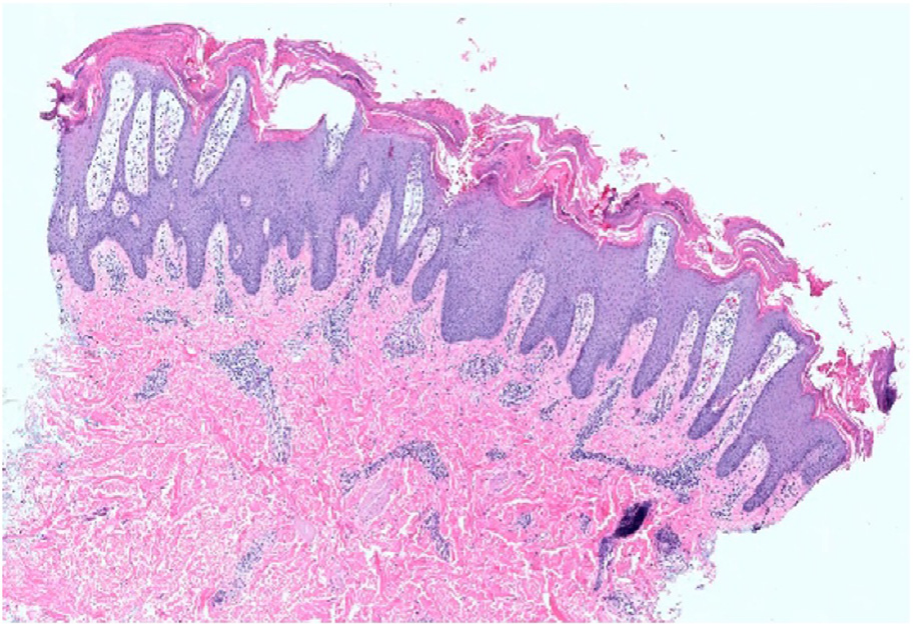
Histopathology of psoriasiform eruption. Biopsy of lesions confirmed the transformation to psoriasiform dermatitis with the presence of psoriasiform epidermal hyperplasia with parakeratosis with neutrophils and a psoriasiform epidermal hyperplasia with dilated blood vessels surrounded by a lymphocytic infiltrate in the papillary dermis consistent with a diagnosis of psoriasis.

The patient’s AD improved on abrocitinib 100 mg daily but due to a flare, his dose was increased to 200 mg daily with subsequent improvement of AD to less than 5% body surface area. After 7 months of treatment, he developed a new cutaneous eruption over his face, chest, and arms consisting of salmon-colored, scaly, well-demarcated papules and plaques affecting approximately 20% body surface area (Fig 2). Morphologically, the eruption was different than the patient’s prior AD in that there were well-demarcated scaling plaques, and the patient denied significant pruritus. Biopsy of this new eruption demonstrated psoriasiform epidermal hyperplasia, parakeratosis with neutrophils, and dilated blood vessels surrounded by a lymphocytic infiltrate in the papillary dermis and was histologically consistent with a diagnosis of psoriasis (Fig 3). Due to the clinical history and biopsy results, a diagnosis of psoriasis secondary to abrocitinib was made. Abrocitinib was subsequently decreased to 100 mg daily resulting in resolution of his psoriasis-like eruption.

## Discussion

AD and psoriasis are believed to be pathologic cutaneous manifestations of 2 separate CD4+ lineages which typically do not coexist. AD is classically associated with dysregulation of the Th2 subset (IL-4, IL-5, IL-13, and IL-31), whereas the Th17 subset (IL-17, IL-21, and IL-22) has been implicated in the pathogenesis of psoriasis.^6^ A progression to psoriasis in a patient with prior AD would suggest a significant alteration in this patient’s cytokine profile from overexpression of Th2 cytokines to overexpression of Th17 cytokines.

Given their opposing immune mechanisms, concomitant cases of AD and psoriasis have previously been thought to be rare. Certain subtypes of AD, including in patients of Asian descent and in those with intrinsic AD and pediatric AD, have been shown to have a predominance of IL-17 expression and histopathologically show overlap with psoriasis.^7^ Due to this, some patients may be more susceptible to developing a phenotypic switch on selective biologic therapy. Multiple instances of a paradoxical reaction of new psoriasis in patients with AD have been described following initiation of dupilumab.^8^ Treatment in such cases is typically a JAK inhibitor as such agents can be effective for both AD and psoriasis given their broader immune blockade that encompasses both Th2 and Th1 cytokines.^4^ For example, upadacitinib, a selective JAK1 inhibitor, achieved complete response in a case series of 4 patients with AD and concomitant psoriasis.^9^

However, this broader blockade may result in aberrant modulation of the inflammatory axis. While some JAK inhibitors, including upadacitinib and abrocitinib, are marketed as being selective, there is evidence that JAK1 inhibitors interact with the JAK2/3 and tyrosine kinase pathways which results in blockade of a broader number of cytokines and explains JAK inhibitor’s ability to treat both psoriasis and AD.^9^^,^^10^ This broader blockade should theoretically protect against phenotypic switching but interestingly in this case did not. In support of this theory, a case has also been reported of a patient with ADwho developed plaque psoriasis on dupilumab which later flared again when he was placed on upadacitinib.^5^

In conclusion, we present a case of a patient with AD who developed psoriasis after initiation of a selective JAK1 inhibitor, abrocitinib. This etiology is supported by the temporal relationship of the new eruption (beginning just after the medication was started/improved after decreasing the dose), change in morphology from his prior AD, and by biopsy results which support a diagnosis of psoriasis. To our knowledge, no other cases of abrocitinib-associated psoriasis have been reported and we hope the inclusion of this case in the literature shows a phenotypic switch may occur despite a broader cytokine blockade. While JAK inhibitors are redefining the treatment landscape of AD, further studies are needed to fully characterize the molecular mechanisms underlying the response to JAK inhibitors to better understand both disease pathogenesis and possible side effects.

## Conflicts of interest

Eric Simpson reports personal fees from AbbVie, Amgen, Arcutis, Areteia Therapeutics, Bristol Myers Squibb–BMS, CorEvitas, Corvus, Dermira, Eli Lilly, Evelo Biosciences, FIDE, Forte Bio RX, Galderma, GlaxoSmithKline, Gilead Sciences, Impetus Healthcare, Incyte, Innovaderm Reche, Janssen, Johnson & Johnson, Kyowa Kirin Pharmaceutical Development, Leo, Merck, NUMAB Therapeutics AG, Pfizer, Physicians World LLC, PRImE, Recludix Pharma, Regeneron, Roivant, Sanofi-Genzyme, SITRYX Therapeutics, Trevi therapeutics, and Valeant. Eric Simpson reports grants (or serves as the principal investigator) for AbbVie, Acrotech, Amgen, Arcutis, ASLAN, Castle, CorEvitas, Dermavant, Dermira, Incyte, Lilly, Kymab, Kyowa Kirin, National Jewish Health, Leo, Pfizer, Regeneron, Sanofi, Target, and VeriSkin. Sarah L. Becker and Adeeb Haroon have no conflicts of interest to declare.
